# Mild Approach for the Formulation of Chestnut Flour-Enriched Snacks: Influence of Processing Parameters on the Preservation of Bioactive Compounds of Raw Materials

**DOI:** 10.3390/foods13172651

**Published:** 2024-08-23

**Authors:** Giovanni Cascone, Maria Oliviero, Luigi Sorrentino, Giuseppina Crescente, Floriana Boscaino, Andrea Sorrentino, Maria Grazia Volpe, Stefania Moccia

**Affiliations:** 1 National Research Council, Institute of Food Sciences, 83100 Avellino, Italy; giovanni.cascone@isa.cnr.it (G.C.); giuseppina.crescente@isa.cnr.it (G.C.); floriana.boscaino@isa.cnr.it (F.B.); mgvolpe@isa.cnr.it (M.G.V.); stefania.moccia@isa.cnr.it (S.M.); 2 National Agency for New Technologies, Energy and Sustainable Economic Development, 80055 Portici, Italy; 3 National Research Council, Institute of Polymers, Composites and Biomaterials, 80055 Portici, Italy; luigi.sorrentino@cnr.it (L.S.); andrea.sorrentino@cnr.it (A.S.)

**Keywords:** plant-based flour, expanded snack, healthy snacking, functional and textural properties, processing parameters, volatile compounds

## Abstract

Third-generation snacks were developed from a triad of flours made up of chestnut, spelt, and chickpea flour. Optimal snack formulations and processing parameters have been established to ensure acceptable workability of the raw dough while protecting the bioactive components of the raw materials. The parameters examined were mixing time, speed, and temperature. The properties of the snack were evaluated by analyzing the expansion ratio, hardness, moisture content, and phenolic and volatile compounds. The optimal mixing conditions that ensure maximum expansion were a temperature of 30 °C, a speed of 30 rpm, and a time of 6 min. The results showed that the proper percentage of water and sodium bicarbonate was 35% and 2%, respectively, and that the developed snacks had an alveolar and homogeneous structure. The proposed approach brings several advantages, including the preservation of bioactive compounds during the production process. Furthermore, the mild operating conditions prevented the development of unwanted or unpleasant compounds, as confirmed by the analysis of volatile compounds. Therefore, this study opens new perspectives in the food industry, satisfying the growing demand for functional products and healthy snacks.

## 1. Introduction

In recent years, a rising part of the population enjoy eating “on the go” foods rather than having formal meals. In response to this, the food industry has developed a wide range of items that can be eaten between meals, called snacks [1]. These products are distinctive, constantly evolving, and ever-expanding [2]. Consumers can choose from a variety of ready-to-eat snacks on grocery store aisles that are available in all sorts of shapes, sizes, colors, and tastes [3].

Snacks are usually made from grains and grain fractions, but the consumer trend towards novel and functional foods has led to the development of products in which wheat is partially or totally replaced by other vegetable flours [4,5]. The introduction of new snack-type functional foods based on plant-based flours, such as those from legumes and cereals, could lead to health benefits, acceptance rates, and higher consumption [6]. The use of plant-based flours results in a higher content of fiber, proteins, and phytochemicals and a lower amount of fat, making them a healthier and alternative food to snacks made from refined flours [4,6].

Phenolic compounds are naturally found in many fruits, vegetables, and grains and can exist in either free or bound forms [7,8]. Usually, bound phenols form covalent bonds with the structural components of the cell wall (cellulose, hemicellulose, lignin, pectin, and structural proteins) [9]. An overwhelming majority of phenolic compounds found in cereal-based matrices are bound to insoluble forms, whereas many soluble conjugates are abundant in fruits and vegetables [10].

Based on composition and processing techniques, snacks are divided into three generations [3]. Snacks falling into the first category encompass items like nuts, potato chips, and popcorn. The second category includes snacks that typically consist of single-ingredient, simple shapes such as corn or tortilla chips, and puffed corn curls and directly expanded snacks. Snacks belonging to the third generation (also known as half-products or pellets) are products formed using multiple ingredients [2]. Such third-generation snacks (TGSs) must undergo an expansion process before consumption as they are not yet ready to eat [11]. Various expansion methods are reported in the scientific literature, including deep-fat frying, hot-air puffing, baking, and infrared or microwave heating [12].

In the food industry, snacks are commonly produced by extrusion, one of the most affordable methods [13]. Products made by extrusion have a lower moisture content, have a longer shelf life, and are microbiologically safe [14,15]. However, several works reported that during extrusion, the loss of bioactive compounds with antioxidant properties can occur [16,17,18]. Raw materials are processed in different ways to produce finished products with excellent sensory and nutritional properties. Besides affecting the physical and chemical properties of products, technological processes may also affect phenolic components [19].

The purpose of this research was to develop expanded snacks with different plant-based flours, using a mild approach, to preserve the nutritional and beneficial properties of the ingredients, often compromised by high-temperature processes.

Many studies have investigated the use of different plant-based flours to produce expanded snacks. For example, recent research has demonstrated the potential of flours derived from legumes, such as chickpeas, lentils, and peas, to produce expanded snacks with improved nutritional value and sensory properties [20]. Other studies have explored the use of pseudo-cereals such as quinoa and amaranth and their blends with other flours to produce expanded snacks with enhanced protein content and digestibility [21].

Here, in detail, a ternary mixture of flours (chestnut, spelt, and chickpea) in different percentages was chosen and subjected to mixing processes capable of producing snacks with low density and good sensory properties. The most difficult challenge in producing such snacks is attaining textural features comparable to those previously established in the marketplace.

Chestnut flour is characterized by high-quality proteins, dietary fiber (10.8%), low amounts of fat (2–4 g/100 g), and a fairly high amount of bioactive compounds, mainly polyphenols. It is rich in starch (50–60 g/100 g), minerals, and vitamins (primarily group B) [22]. Chickpea is a grain legume, and its flour has a high protein, lipid, and fiber content, as well as a lower carbohydrate content than wheat flour [23,24]. However, chestnut and chickpea-derived products lack gluten [25,26]; for this reason, the functional role of gluten was carried out by spelt flour, a more digestible and low-calorie cereal than wheat, with a high protein and fiber content [27].

Considering this evidence, the proposed work is intended to investigate the optimal mixing conditions in terms of temperature, screw speed, and mixing time, as well as the type and content of the blowing agent. The structural characteristics of the snack and the effects of the entire process on the phenolic content, antioxidant properties and volatile organic compounds of the TGSs enriched with a blend of flours were monitored over time.

## 2. Materials and Methods

### 2.1. Ingredients, Chemicals, and Reagents

Spelt, chickpea, and chestnut flours were purchased from Molini Agostini S.r.l. (Ascoli Piceno, Italy); all the flours were wholemeal. Typical flour compositions can be found in the Supplementary Materials. Sodium bicarbonate (Solvay, Livorno, Italy), instant yeast (Paneangeli, Desenzano del Garda, Italy), fine salt (Italkali–Società Italiana Sali Alcalini S.p.A., Palermo, Italy), and potable water were acquired from retail stores. Ethanol (EtOH), methanol (MeOH), ethyl acetate (EtOAc) (analytical grade), and hydrochloric acid 37% (*v*/*v*) were purchased from Carlo Erba Reagents (Milan, Italy). Other reagents were purchased from Merck-Sigma (Milan, Italy).

### 2.2. Thermogravimetric Analysis (TGA) of Flours

The effective moisture content of each flour was evaluated using TGA performed on the Q500 thermogravimetric analyzer (TA-Instruments Corp.; New Castle, DE, USA) under a nitrogen gas purge of 25 mL/min. A sample of about 10 mg of powder was loaded, distributed, and flattened into a flame-cleaned platinum pan and heated at a rate of 10 °C/min from room temperature to 150 °C. Moisture content was determined as weight loss at 120 °C using Universal Analysis 2000 software (version 4.5) from TA-Instruments. All measurements were conducted in triplicate.

### 2.3. Preparation of Expanded Snacks-Experimental Design

The control formulation was composed of chestnut flour (12.5 g), spelt flour (32 g), chickpea flour (17.5 g), salt (1 g), sodium bicarbonate (2 g), and potable water (35 g) (all in g/100 g of dough). The effects of three independent mixing parameters (variables), mixing temperatures (*T)*, screw speed (*v*), and mixing times (*t*) on the expansion ratio of the products were studied to optimize the processing parameters. A total of 20 snack samples using the control formulation were prepared. All the ingredients were mixed in a beaker using a spatula to obtain a crude mixture. The mixture was then fed into a twin counter-rotating internal mixer (Rheomix^®^ 600, Thermo Haake; Karlsruhe, Germany) equipped with roller rotors and connected to a control unit (Polylab QC, Thermo Haake; Karlsruhe, Germany). The mixing chamber was first filled with 80 g of total mass for all experiments in approximately 3 min at 5 rpm at different mixing temperatures, *T*. Subsequently, the rotation speed, *v*, was increased and maintained at different rpm values for various mixing times, *t*. The test details are presented in Table 1. The torque and temperature of the dough were recorded during the mixing process to determine the history of the mixing process.

A pasta rolling machine was used to roll out the dough extracted from the mixer into thin sheets (approximately 3.3 mm thick), from which dough discs with a diameter of 39 mm were cut. Next, the circular samples were cooked and expanded simultaneously in a ventilated oven (model G3 G10073, Ferrari S.r.l.; Rimini, Italy) operated at 180 °C for 10 min to produce the snacks. Finally, the expanded snacks were left at room temperature for 60 min and then stored in plastic bags. All snacks were prepared in triplicate.

Once optimized the mixing parameters, other snacks were developed in these conditions with different amounts of water (30, 33.5 and 35 g/100 g of dough), sodium bicarbonate (0, 1.6, 3.2, 4.8, and 6.1 g/100 g of dough), and instant yeast (0, 1.6, 3.2, 4.8, and 6.1 g/100 g of dough). Also, for the determination of snack formulation, the product property used was the expansion ratio.

### 2.4. Characterization of Expanded Snacks

#### 2.4.1. Snack Density and Expansion Degree

Snack density (*SD*) was determined as the ratio of weight (measured with an analytical balance) to volume (measured with a high-resolution caliper) of the sample. The degree of expansion (*RD*) was calculated as the ratio of the densities of the snack sample before (*SD_COOKED_*) and after the cooking process (Equation (1)):

(1) $$RD=\frac{{SD}_{COOKED}}{SD_{CRUDE}}*100$$

#### 2.4.2. Microstructure

Optical imaging was performed at different magnifications (1×, 2×, and 4×) on selected samples by means of a stereomicroscope (model ZA16 APO, Leica Microsystems GmbH; Wetzlar, Germany) to qualitatively check the cellular morphology of the snacks after baking.

#### 2.4.3. Extraction of Phenolic Compounds

Free (FP) and bound phenolic compounds (BP) were extracted from the flour mixture (in the same ratio reported in Section 2.3), doughs, and snacks following the method reported by Martín-Diana et al. [28]. 1 g of each sample was mixed with 20 mL of cold EtOH/H_2_O (80:20, *v*/*v*), and extraction was performed at room temperature by magnetic stirring for 10 min. Two extraction cycles were performed. At the end of each cycle, the sample was centrifuged (Neya 16R; 2500 rpm for 10 min at 25 °C), the supernatant was filtered, and the extraction solvent was removed using a rotary evaporator (Heidolph Hei-VAP Advantage; Schwabach, Germany). The extract was redissolved in 10 mL of MeOH/H_2_O (80:20, *v*/*v*). To recover BP, the residue was treated with 12 mL of distilled water (H_2_O_d_) and 5 mL of 10 M NaOH and mixed at room temperature overnight using a magnetic stirrer. The pH of the solution was adjusted to 2, and the released phenolic acids were extracted with 15 mL of EtOAc and centrifuged (12000 rpm for 10 min at 25 °C). The extraction cycle was repeated thrice, and the supernatants were pooled. Following alkaline hydrolysis, 2.5 mL of concentrated HCl was added to the residue, which was then incubated in a water bath for 30 min at 85 °C. After cooling, EtOAc was used to extract the phenolic compounds. Organic fractions from alkaline and acid hydrolysis were blended, evaporated using a rotary evaporator, and redissolved in 10 mL of MeOH.

#### 2.4.4. Determination of Total Phenol Content (TPC)

TPC was determined in the free and bound fractions by the Folin–Ciocalteu method, as reported by Cicco et al., with slight modifications: 0.1 mL of samples were mixed with 2.3 mL of H_2_O_d_, 0.1 mL of Folin–Ciocalteu reagent (diluted 1:1 with H_2_O_d_, *v*/*v*), and 0.2 mL of Na_2_CO_3_ (20%, *w*/*v*) [29]. The tubes were mixed and left to react for 90 min in the dark at room temperature. The absorbance was read at 760 nm by transferring 1 mL of each sample into cuvettes (DU730 UV-Vis Spectrophotometer, Beckman Coulter; Milan, Italy). Phenolic content was quantified using a calibration curve of gallic acid standard solution. Data were expressed as milligrams of gallic acid equivalents (GAEs) per 100 g of extract.

#### 2.4.5. Determination of ABTS Radical Scavenging Capacity

The free and bound phenolic fractions were tested toward 2.2′-azinobis-(3-ethylbenzothiazolin-6-sulphonic acid (ABTS). ABTS was prepared as previously reported by Crescente et al. [30]. Subsequently, the solution was diluted in Phosphate-Buffered Saline (PBS; pH 7.4) to obtain an absorbance of 0.7 at 734 nm. The extracts (0.1 mL) were added to 2.9 mL of the ABTS^●+^ solution. After incubation (6 min), the absorbance was measured at 734 nm (DU 730, Beckman Coulter; Milan, Italy) in reference to a blank. Trolox was used as a positive standard. The results were expressed in terms of the percentage decrease in the initial ABTS^●+^ absorption by the test samples.

#### 2.4.6. Volatile Molecules by Gas Chromatography/Mass Spectrometry

The volatile fraction of doughs and snacks was analyzed with headspace sampling using the solid-phase microextraction technique (HS-SPME) according to Reale et al. with modification [31]. For each SPME analysis, 2 g of samples were placed into a 20 mL headspace vial and added of 5 µL of 3-octanol (internal standard, 100 mg/L standard solution). The vial was placed in a thermostatic block (40 °C) on a stirrer, and after equilibration, the headspace of the samples was sampled using an SPME fiber coated with DVB/CAR/PDMS (divinylbenzene/carboxen/polydimethylsiloxane, thickness 50/30 mm) at 40 °C for 30 min. The HS-SPME analysis was performed with an Agilent GC 7890A/MSD 5975 system with an automatic sampler Gerstel MPS2 (Agilent Technologies, Santa Clara, USA). The operating conditions were as follows: HP-Innowax capillary column (Agilent Technologies, 30 m × 0.25 mm ID, film thickness 0.25 μm), the gas carrier was helium (flow 1.5 mL/min), and SPME injections were splitless (straight glass line, 0.75 mm I.D.) at 240 °C for 10 min, during which time thermal desorption of analytes from the fiber occurred. The oven parameters were as follows: the initial temperature was 40 °C held for 3 min, followed by an increase to 240 °C at a rate of 5 °C/min, and then held for 5 min. The injector temperature was 240 °C. The mass spectrometer operated in scan mode over the mass range from 33 to 300 amu (2 s/scan) at an ionization potential of 70 eV. Volatile organic compounds (VOCs) were identified based on their mass spectra using the Wiley library (Wiley7, NIST 05). The data were expressed in a relative peak area with respect to internal standards. Blank experiments were conducted in two different modalities: the blank of the fiber and the blank of the empty vial. All analyses were performed in triplicate.

#### 2.4.7. Storage Studies

Snack samples of about 4 g were stored in a desiccator at 70% relative humidity (RH) and room temperature using a saturated salt solution of potassium iodide (KI). This humidity value was chosen to simulate typical snack storage conditions. Samples were withdrawn at predetermined intervals, weighed to determine moisture content, and subjected to textural measurements to determine change in product quality during storage. All analyses were performed in triplicate.

##### Moisture Content

The moisture content of the snack samples, stored at room temperature and 70% relative humidity, was determined using a static gravimetric technique. The samples taken from the desiccators were weighed periodically with an analytical balance (PCB-1000, Kern & Sohn GmbH; Balingen, Germany) for approximately two weeks. The tests were performed in triplicate, and the evolution of water uptake (*WU*) over time was determined according to Equation (2):

(2) $$WU(t)=\frac{1}{3}*\sum_{I=1}^{3}\frac{\left(Mt,i-M0,i\right)}{M0,i}$$

where *M_t_* and *M_0_* are the current and the initial weights of the samples, respectively.

##### Texture Measurement

The hardness of the snack samples was measured using a universal testing machine (SANS CMT 4304, Sans Materials Testing Co.; Shenzen, China) equipped with a blade/plate system to simulate the functionality of the texture analyzer for this type of food. The instrument was equipped with a 250 N load cell, typical of a texture analyzer. The cross-head speed was set to 5 mm/min. The samples were punctured using the probe up to 20 mm, corresponding to approximately 50% of the diameter of the snacks. All samples had a thickness of 3 mm and a diameter of 40 mm. The hardness in N was determined by measuring the maximum force required to break the snack samples. The test speed was 5 mm/min. To evaluate the effect of storage on the hardness of snacks, tests were performed on snacks taken from desiccators at different times. Ten measurements were performed for each snack sample, and their average was taken as the mean value.

### 2.5. Statistical Analysis

The data were expressed as mean values ± standard deviation (SD) on three independent measurements (*n* = 3). For TPC and ABTS, the significance was measured using the Student’s test; values with *p* < 0.05 were considered statistically significant.

## 3. Results and Discussion

### 3.1. Optimization of Mixing Parameters and Recipe

**Figure 1:**
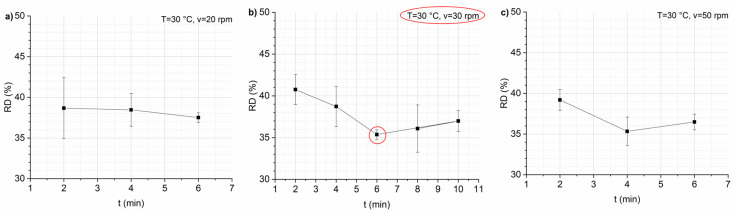
Percentage of expansion ratio (RD, %) vs. mixing time *t* for each screw speed *v*: 20 (**panel a**), 30 (**panel b**), and 50 rpm (**panel c**). Data are expressed as mean ± SD (*n* = 3).

To achieve the lowest RD, various preliminary mixing tests were conducted to optimize the process variables (*T*, *v*, and *t*). RD is one of the main quality indicators of expanded snacks; it determines the depth of physicochemical transformations of nutrients during the mixing of raw materials containing starch [32]. The RD values calculated at 30 °C and different *t* for each *v* are reported in Figure 1. The results show that a minimum RD value was found both at 30 °C, 30 rpm, and 6 min and at 30 °C, 50 rpm, and 4 min. However, the smallest standard deviation of the values was detected at 30 °C, 30 rpm, and 6 min. This implies that the snacks produced under these conditions are more uniform, and the dough obtained is more homogeneous compared with that obtained at 30 °C, 50 rpm, and 4 min.

**Figure 2:**
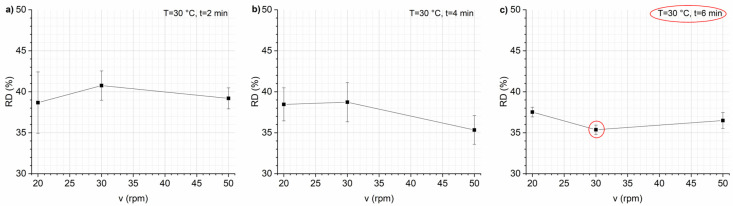
Percentage of expansion ratio (RD, %) vs. screw speed *v* for each mixing time *t*: 2 (**panel a**), 4 (**panel b**), and 6 (**panel c**). Data are expressed as mean ± SD (*n* = 3).

Mixing time has an interesting effect on RD (Figure 1). This effect is related to the speed of the screw. For a *v* of 20 rpm, the RD decreases slightly with mixing time, whereas for higher *v* (30 and 50 rpm), the RD first decreases and then increases with time, probably due to the excess development of gluten, which occurs when mixing the dough for longer times. This causes the dough to become very elastic, resulting in reduced expandability [33]. The higher the speed, the shorter the RD minimum time (6 min at 30 rpm and 4 min at 40 rpm). Screw speed was observed to have a significant effect on RD, as shown in Figure 2. Increasing screw speed caused a decrease in RD. Launay and Lisch reported that the higher shear resulting from increased screw speed reduces the melt viscosity of the dough, which in turn results in a decrease in RD [34]. The same considerations are confirmed by other authors [35,36].

Finally, the tests conducted at 50 °C revealed an increase in RD at all mixing times and screw speeds compared with those obtained at 30 °C. A similar effect of temperature on expansion was found by Thakur et al. [37]. An inverse relationship between extrusion temperature and expansion ratio was reported by Charunuch et al., Kaur et al., and Alam et al. [38,39,40]. In these studies, the expansion significantly increased with increasing extrusion temperature and was attributed to its higher degree of gelatinization. This indicates that the effect of a higher temperature might be positive or negative on the expansion characteristic depending on the compositions and rheological properties of the input materials [41].

**Figure 3:**
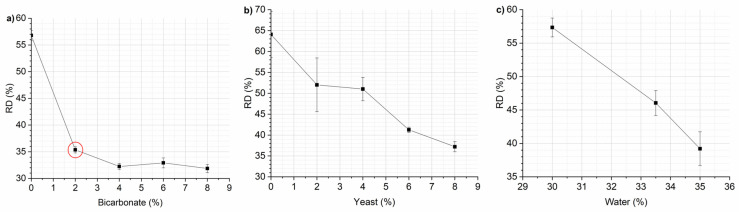
Percentage of expansion ratio (RD, %) vs. leaving agent content: bicarbonate (**panel a**), yeast (**panel b**), and water (**panel c**). Data are expressed as mean ± SD (*n* = 3).

After determining the optimal mixing parameters, such as 30 °C, 30 rpm, and 6 min, the investigations proceeded by varying the amount of the expanding agent, specifically bicarbonate. The graph of RD vs. bicarbonate amount revealed that an increase in bicarbonate results in a decrease in RD (Figure 3, panel a).

Albeit this could be a beneficial effect, the increase in bicarbonate affects the taste of the product. Consequently, the optimal percentage of expanding agent was set to 2% since higher quantities of it imparted the snack a bitter aftertaste with a negligible increase in expansion. A small increase in the expansion ratio was preferred to preserve the flavor of the product.

Alternatively, instant yeast was also tested as a leavening agent but yielded lower expansion results compared with bicarbonate, as shown in Figure 3 (panel b). Since the water present in the dough also acts as a leavening agent, the effect of different water contents (30, 33.5, and 35 wt%) on RD was also evaluated (Figure 3, panel c). In this case, it is necessary to consider the restrictions imposed by the thermoplastic mixing process to obtain expanded snacks with a correctly developed porous structure and suitable RD. The process limits the moisture content of the recipe mix (10–40%). At higher humidity, the amount of applied thermal energy is not enough for the needed fast evaporation of moisture from starch grains, while at lower humidity, the amount of steam formed during processing is not enough to completely break the starch grains. Thus, in both cases, the porosity of the snack is poorly developed and unevenly distributed [32]. For bicarbonate and instant yeast, the RD was reduced with an increase in water content. As optimal water content, 35 wt% of water was considered for the final snack recipe.

**Figure 4:**
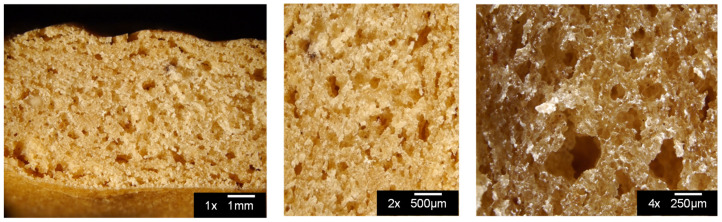
Optical images at different magnifications (1×, 2× and 4×) of a cross-section of snacks produced at 30 °C, 30 rpm, and 6 min with 2 wt% bicarbonate and 35 wt% water.

The optical images, reported in Figure 4, of a cross-section of snack produced at 30 °C, 30 rpm, and 6 min with 2 wt% bicarbonate and 35 wt% water highlighted high porosity determined by the presence of numerous small bubbles well distributed in the sample and a few large bubbles. These processing parameter values were used as reference conditions for the following characterizations.

### 3.2. Processing Influence on TPC and Antioxidant Activity

**Figure 5:**
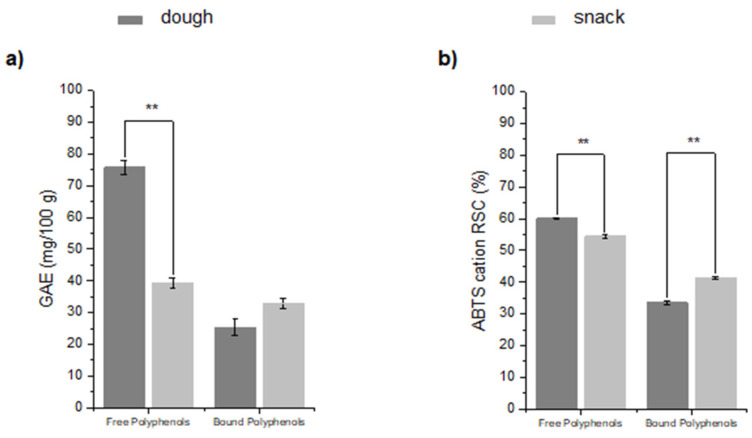
Changes in phenolic contents (**panel a**) and antioxidant activity (**panel b**) of free and bound polyphenols in dough and in snacks after baking processes. Data are expressed as mean ± SD (*n* = 3). Symbols indicate significance: ** *p* < 0.01 dough vs. snack (Student’s *t*-test).

Food preparation, whether commercial or domestic, involves a wide variety of processes [42]. Few studies have been conducted to investigate how baking affects phenolic compounds in bakery products, both free and bound compounds [43]. The raw materials used in this study are a good source of phenolic compounds, but the amount of phenolics in the final products can vary significantly. Panels a of Figure S1 and Figure 5 show the content of free and bound polyphenols in the flour mix, in the dough and in the final snack: the two fractions exhibit a different trend depending on the processing phase. From the flour mix to the dough, a small reduction (10.7%) in total polyphenol content was observed: the dough undergoes a mixing process that ensures that the ingredients are evenly distributed and blended. During high-speed mixing, disulfide bonds in proteins break, creating thiol free radicals and reducing compounds; furthermore, the oxidative enzymes found in flour, including oxygenase and peroxidase, are hydrolyzed. Following the formation of new bonds between phenols and proteins, the phenolic content may decrease due to a reduction in their bioaccessibility and, hence, extractability [19].

Regarding the leavening and fermentation process, the studies conducted on the phenol content are inconsistent: despite this, fermentation conditions, specifically temperature, pH, and duration, play a pivotal role. For example, a study reports that when fermentation takes place for a long time, many bonds between phenolic acids and fibers are broken, increasing their bioavailability [44]. Likewise, the fermentation with sourdough increases the release of insoluble bound phenolic compounds by lowering pH [45].

Lastly, baking is considered the most important stage in which physical, chemical, and biochemical changes occur due to the exchange of heat and material [19]. The baking time does not appear to have an impact [46]; instead, a high temperature causes thermolabile phenolics to decompose, resulting in the loss of antioxidants. Furthermore, during baking, new phenolic structures can be generated because of the Maillard reaction [19].

According to the cited studies, our samples showed a reduction in the free polyphenol fraction after cooking (*p* < 0.01), whereas the amount of bound phenolics increased in comparison to the dough. These results agree with some studies that claim baking destroys phenolic compounds [47]. Holtekjølen et al. investigated the effect of baking and storage on the antioxidant properties of bread containing different barley flours: a decrease in free phenolics was observed during the baking process, while bound phenolics increased [48]. Another study conducted by Ross et al. evaluated the effect of heating on TPC and antioxidant activity in grape seed flour, a waste product generated from winemaking. After 180 °C heating for 10 min, TPC was significantly lower (*p* < 0.05) than the unheated control, suggesting that thermal degradation was the predominant mechanism responsible for its reduction [49]. Moreover, Alfeo et al. explored the effects of different baking programs on the production of bakery products made from fresh wheat sprouts: during baking, the bound polyphenols were released [50].

Similarly, the antioxidant activity was investigated in the flour mix before the baking process (dough) and after (snacks). The antioxidant activity remained relatively stable throughout the entire process despite the increase in temperature during the baking process (Figure S1 and Figure 5, panels b). The decrease in polyphenol content did not correspond to a decrease in antioxidant activity. A similar result was obtained by Holtekjølen et al., who found that antioxidant activity remained relatively stable after baking [48]. Moreover, polyphenols released from food matrixes are more bioavailable and easier to absorb in the intestinal tract [50]. The nutraceutical properties of polyphenols have made them popular in bakery foods.

### 3.3. Volatile Compounds Determination

HS-SPME-GC/MS analysis revealed the presence of a total of 44 VOCs (Table 2), classified into eight classes: alcohols (10 compounds), aldehydes (8 compounds), terpenoids (8 compounds), ketones (5 compounds), acids (5 compounds), pyrazines (4 compounds), esters (2 compounds), and furans (2 compounds). Alcohols are the most abundant volatile compounds in the dough. In fact, by summing the peak areas of the volatiles in the dough, alcohols account for 51.1% of peak areas, followed by aldehydes (31.6%), terpenoids (6.1%), ketones (5.7%), and other volatiles present at very low levels (<2%). On the contrary, the most volatile compounds in the snack were aldehydes (35.8%), followed by alcohols (28.9%), esters (17.3%), ketones (7.6%), terpenoids 6.8%, and other volatiles occurring at very low levels (<2%).

The main differences between the dough and the snack were a decrease in alcohol and an increase in esters. The compounds in the snack originated from the Maillard reaction and lipid oxidation. Non-enzymatic Maillard reactions involve the reaction between amino acids and sugars, leading to the formation of brown pigments (melanoidin) and many volatile compounds like (2-pentylfuran), 2-furanmethanol, and pyrazine derivatives [51]. No unwanted or unpleasant compounds were observed in the snack samples.

### 3.4. Changes in the Moisture Content and Hardness of Expanded Snacks during Storage

Moisture content represents one of the most important parameters for a snack. Its content affects the texture, taste, mouthfeel, appearance, stability, and shelf life of the snack [52]. Excess humidity can affect the creak by making the item stale or, even worse, contributing to the growth of bacteria/mold [53]. Moisture content has a direct effect on the way it is processed, blended, and dried. Having too much moisture can make the viscosity of the dough too low and create lumps in dry mixes. For all the above, moisture content must be monitored during the processing, mixing, and drying of a snack [54].

**Figure 6:**
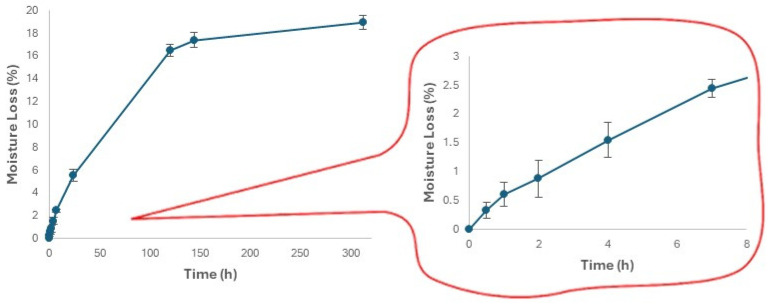
Moisture loss of the snack sample as a function of storage time at HR = 70%. Data are expressed as mean ± SD (*n* = 3).

Since there is no standard storage condition for all products, it is necessary to determine the optimal storage condition for the specific product. In the present case, room temperature and 70% relative humidity were chosen to replicate home storage conditions. The moisture loss from the snack samples reported in Figure 6 was determined as the mass loss of the conditioned samples according to Equation (2). The moisture loss was steadily growing until around 144 h (6 days) when it reached a value of 18% (Figure 6). Usually, in literature, the authors report an increase in moisture content in ready-to-eat extruded snacks during storage [55,56,57]. In this work, the continuous loss of moisture from the product throughout the storage period can be correlated with the nature and porous structure of the product and the storage environment (temperature, relative humidity) [58].

**Figure 7:**
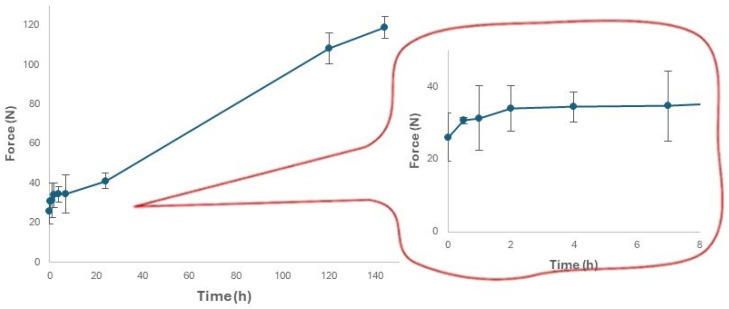
Hardness of the snack sample as a function of the storage time at HR = 70%. Data are expressed as mean ± SD (*n* = 3).

The loss of moisture during storage was also evident in the progressive hardening of the snack and the corresponding loss of its flavor. Snack stiffness is defined in the present paper as the maximum force required for a probe to penetrate the snack [59]. The change in stiffness of the snack during the storage period is represented in Figure 7. The mean value reflects a gradual increase over the entire storage duration. The lowest mean value of stiffness was 25.95 ± 6.65 N at 0 h, and the highest mean value was 118.67 ± 5.56 at 144 h.

Previous studies found the same impact of moisture content on stiffness, relating it to the occurrence of different events in short and long storage times, and is likely related to the structural conformation of starch and its interaction with water [60,61]. Gelatinized starch is known to be subjected to amylopectin retrogradation and staling during storage, resulting in increased stiffness. A commercial snack should be stored at room temperature for at least one week without losing mouthfeel and texture. For this reason, it is necessary to ensure a sufficient water content. One possible strategy is to add moisture and keep it constant within the snack package using adequate water vapor permeation barrier films, but this must be performed without increasing water activity. The high activity of water, in fact, accelerates the oxidation of lipids and favors microbial growth and the development of mold. Water activity can be reduced by adding solutes such as salt or sugar, which help to extend the shelf life of the snack, but this can affect the processability and cookability of the dough; hence, a careful balance between the different elements must be identified.

## 4. Conclusions

The growing demand for healthier snack options required identifying innovative ingredients and processes capable of enhancing and preserving both physical and nutritional properties. In this study, the third-generation snacks (TGSs) were developed from a triad of flours made up of chestnut, spelt, and chickpea flour using a mild approach based on a low-temperature mixing process and subsequent baking in the oven. The research focused on identifying the optimal processing parameters, type, and content of blowing agents that result in snacks with a high degree of expansion, monitoring the textural characteristics of snacks during storage, and analyzing the effects of the proposed mild approach on the phenolic content, antioxidant properties, and volatile organic compounds of TGSs.

The findings showed that the optimal mixing conditions that ensure maximum expansion are a temperature of 30 °C, a speed of 30 rpm, and a time of 6 min. Under these conditions, the dough had adequate elasticity and degree of gelatinization, which improved its expandability. Considering the blowing agent, baking soda was more efficient than instant yeast, and the proper percentages of water and baking soda were 35% and 2%, respectively. As a result, the developed snacks had a homogeneous and alveolar structure, determined by the presence of numerous small bubbles well distributed in the sample and a few large bubbles. Furthermore, the parameters used allowed the safeguarding of the bioactive compounds, particularly the bound polyphenols with good bioavailability, during the production process, and the mild operating conditions prevented the development of unwanted or unpleasant compounds, as confirmed by the analysis of volatile compounds.

To the best of our knowledge, this is the first study highlighting the potential of a mild laboratory-scale approach in the development of expanded healthy snacks enriched with preserved bioactive compounds. From future perspectives, we will evaluate the implementation of mild conditions in an extrusion process to study the scalability of the proposed approach for the food industry, satisfying the growing demand for functional products and healthy snacks.

## Figures and Tables

**Table 1:** Melt mixing parameters.

Test Number	*T* (°C)	*v* (rpm)	*t* (min)
1	30	20	6
2	30	20	4
3	30	20	2
4	30	30	6
5	30	30	4
6	30	30	2
7	30	50	6
8	30	50	4
9	30	50	2
10	50	20	6
11	50	20	4
12	50	20	2
13	50	30	6
14	50	30	4
15	50	30	2
16	50	50	6
17	50	50	4
18	50	50	2
19	30	30	8
20	30	30	10

**Table 2:** Volatile organic compounds (VOCs) identified in dough and in snacks after baking processes. Data are expressed in relative peak area (Area peak compound/Area peak Internal Standard × 100). The values reported are the mean ± SD (*n* = 3).

	Dough	Snack
***Aldehydes***		
2-methylbutanal	8.2 ± 0.4	nd
3-methylbutanal	19.9 ± 1.8	nd
Hexanal	1543.7 ± 124.1	1607.2 ± 18.6
Nonanal	3.0 ± 0.2	nd
2-octenal	6.2 ± 0.1	nd
2-Nonenal, (Z)-	4.0 ± 0.4	nd
Benzaldehyde	10.1 ± 0.6	1.2 ± 0.0
Octanal	4.9 ± 0.5	nd
**Total**	**1600.0 ± 126.6**	**1608.4 ± 18.6**
***Alcohols***		
Ethyl alcohol	1560.7 ± 81.9	1258.7 ± 11.4
1-Penten-3-ol	217.8 ± 0.1	nd
isoamyl alcohol	83.9 ± 4.4	nd
1-Pentanol	188.8 ± 9.0	nd
2-Penten-1-ol, (Z)	139.4 ± 1.3	6.5 ± 0.4
1-Hexanol	498.9 ± 2.9	27.9 ± 0.3
3-Hexen-1-ol, (z)	3.5 ± 0.1	nd
1-octen-3-ol	31.2 ± 0.8	1.8 ± 0.1
Benzyl Alcohol	4.4 ± 0.1	2.6 ± 0.2
Phenylethyl Alcohol	7.4 ± 0.4	3.6 ± 0.3
**Total**	**2736.1 ± 63.8**	**1301.0 ± 11.6**
***Ketones***		
2-Propanone	249.0 ± 19.3	287.1 ± 17.0
2,3-pentanedione	9.0 ± 0.3	nd
2-heptanone	nd	22.6 ± 1.4
3-hydroxy-2-butanone	21.7 ± 1.6	32.8 ± 1.6
6-methyl-5-hepten-2-one	6.5 ± 0.6	nd
**Total**	**286.2 ± 18.1**	**342.6 ± 20.1**
***Esters***		
Ethyl Acetate	96.7 ± 9.9	758.5 ± 27.0
Isoamyl acetate	nd	20.3 ± 0.5
**Total**	**96.7 ± 9.9**	**778.8 ± 26.5**
***Acids***		
Acetic acid	12.4 ± 0.7	4.5 ± 0.4
Hexanoic acid	nd	3.7 ± 0.2
Heptanoic acid	nd	6.4 ± 0.4
Nonanoic acid	nd	8.5 ± 0.7
**Total**	**12.4 ± 0.7**	**23.2 ± 0.9**
***Terpenoids***		
Limonene	243.9 ± 18.4	274.8 ± 6.4
Eucalyptol	23.9 ± 0.5	nd
g-terpinene	14.6 ± 0.7	18.5 ± 2.1
o-cymene	5.6 ± 0.1	7.8 ± 0.6
Camphor	2.3 ± 0.2	nd
Linalool	15.3 ± 0.3	5.5 ± 0.3
4-terpineol	1.2 ± 0.1	nd
verbenone	1.7 ± 0.1	nd
**Total**	**308.4 ± 20.1**	**306.5 ± 4.0**
***Pyrazines***		
2,5-dimethyl-pyrazine	nd	42.1 ± 2.8
2,6-dimethyl-pyrazine	nd	16.8 ± 1.1
2,3-dimethyl-pyrazine	nd	1.6 ± 0.1
2-ethyl-5-methyl-pyrazine	nd	9.3 ± 0.9
**Total**	**nd**	**69.8 ± 5.0**
***Furans***		
2-pentyl-furan	19.2 ± 1.5	38.9 ± 3.3
2-Furanmethanol	1.9 ± 0.1	3.0 ± 0.0
**Total**	**21.2 ± 1.6**	**41.9 ± 3.3**

## Data Availability

The original contributions presented in the study are included in the article/Supplementary Materials; further inquiries can be directed to the corresponding author.

## Supplementary Materials

The following supporting information can be downloaded at https://www.mdpi.com/article/10.3390/foods13172651/s1, Table S1: Typical composition of spelt flour; Table S2: Typical composition of chickpea flour; Table S3: Typical composition of chestnut flour; Figure S1. Phenolic contents (panel a) and antioxidant activity (panel b) of free and bound polyphenols in the flour mix. Data are expressed as mean ± SD (*n* = 3).
