# Supplementary material for: Mild Approach for the Formulation of Chestnut Flour-Enriched Snacks: Influence of Processing Parameters on the Preservation of Bioactive Compounds of Raw Materials
Source: Foods. 2024 Aug 23;13(17):2651. doi: 10.3390/foods13172651 (PMC11394262; doi:10.3390/foods13172651)
Supplement: Supplementary file 1 [file foods-13-02651-s001.zip › foods-3148117-supplementary.pdf]

Supplementary materials

The typical compositions of spelt, chickpea and chestnut flours are reported in Table S1, S2 and S3 respectively. While the fat, carbohydrate, fiber, protein, and salt content were taken from the flour data sheet, the effective moisture content of each flour was determined by thermos-gravimetric analysis. The moisture content of flours was not reported in data sheet, but it is an important parameter for the snack formulations.

Table S1. Typical composition of spelt flour.

Composition	g/100g
Fats (Saturated fatty acids)	1.6 (0.3)
Carbohydrates (Sugars)	74 (4.3)
Fibers	3.2
Proteins	14
Salt	0.001
Moisture	8.2

Table S2. Typical composition of chickpea flour.

Composition	g/100g
Fats (Saturated fatty acids)	4.7 (0.63)
Carbohydrates (Sugars)	54 (3.7)
Fibers	13
Proteins	22
Salt	0.001
Moisture	8.8

Table S3. Typical composition of chestnut flour.

Composition	g/100g
Fats (Saturated fatty acids)	2.1 (0.7)
Carbohydrates (Sugars)	77.3 (24.5)
Fibers	3.3
Proteins	5.9
Salt	0.2
Moisture	6.1

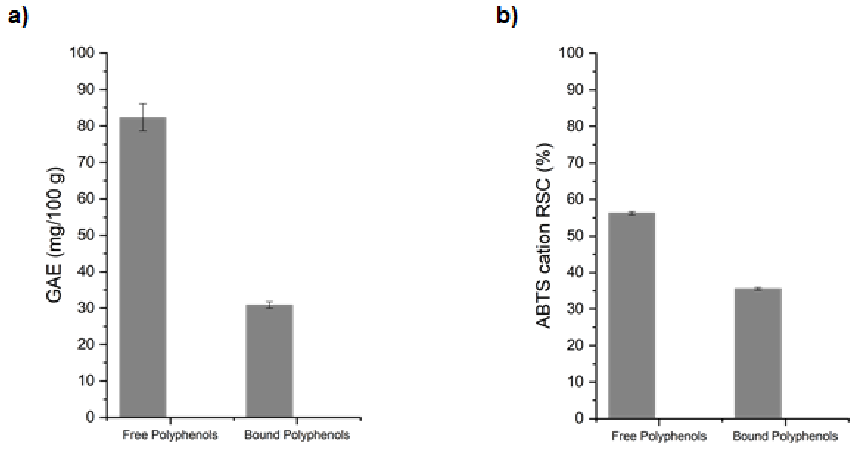

Figure S1. Phenolic contents (panel a) and antioxidant activity (panel b) of free and bound polyphenols in the flour mix. Data are expressed as mean ± SD (n = 3).
